# Role of Positron Emission Tomography in Primary Central Nervous System Lymphoma

**DOI:** 10.3390/cancers14174071

**Published:** 2022-08-23

**Authors:** Laura Rozenblum, Caroline Houillier, Carole Soussain, Marc Bertaux, Sylvain Choquet, Damien Galanaud, Khê Hoang-Xuan, Aurélie Kas

**Affiliations:** 1 Service de Médecine Nucléaire, Hôpitaux Universitaires Pitié-Salpêtrière Charles Foix, AP-HP, Sorbonne Université, 75013 Paris, France; 2 Laboratoire d’Imagerie Biomédicale, INSERM U1146, 75006 Paris, France; 3 ICM, et IHU, Service de Neurologie 2, Hôpitaux Universitaires Pitié-Salpêtrière Charles Foix, AP-HP, Sorbonne Université, 75013 Paris, France; 4 Service d’Hématologie, Institut Curie, Site Saint-Cloud and INSERM U932 Institut Curie, Université PSL, 75005 Paris, France; 5 Service de Médecine Nucléaire, Hôpital Foch, 92150 Suresnes, France; 6 Service d’Hématologie Clinique, Hôpitaux Universitaires Pitié-Salpêtrière Charles Foix, AP-HP, Sorbonne Université, 75013 Paris, France; 7 Service de Neuroradiologie, Hôpitaux Universitaires Pitié-Salpêtrière Charles Foix, AP-HP, Sorbonne Université, 75013 Paris, France

**Keywords:** PCNS lymphoma, ^18^F-FDG PET, outcome, therapeutic evaluation

## Abstract

**Simple Summary:**

Primary central nervous system lymphoma (PCNSL) is a rare but highly aggressive lymphoma with increasing incidence in immunocompetent patients. To date, the only established biomarkers for survival are age and functional status. Currently, the magnetic resonance imaging (MRI) criteria of the International Collaborative Group on Primary Central Nervous System Lymphoma are the only ones recommended for follow-up. However, early occurrence of recurrence after treatment in patients with a complete response on MRI raises the question of its performance in assessing residual disease. While the use of ^18^F-fluorodeoxyglucose body positron emission tomography for identification of systemic disease has been established and can be pivotal in patient treatment decisions, the role of brain PET scan is less clear. Here we review the potential role of PET in the management of patients with PCNSL, both at diagnosis and for follow-up under treatment.

**Abstract:**

The incidence of primary central nervous system lymphoma has increased over the past two decades in immunocompetent patients and the prognosis remains poor. A diagnosis and complete evaluation of the patient is needed without delay, but histologic evaluation is not always available and PCNSL can mimic a variety of brain lesions on MRI. In this article, we review the potential role of ^18^F-FDG PET for the diagnosis of PCNSL in immunocompetent and immunocompromised patients. Its contribution to systemic assessment at the time of diagnosis has been well established by expert societies over the past decade. In addition, ^18^F-FDG provides valuable information for differential diagnosis and outcome prediction. The literature also shows the potential role of ^18^F-FDG as a therapeutic evaluation tool during the treatment and the end of the treatment. Finally, we present several new radiotracers that may have a potential role in the management of PCNSL in the future.

## 1. Epidemiology

Primary central nervous system lymphoma (PCNSL) is a highly aggressive non-Hodgkin’s lymphoma confined to the central nervous system (CNS), including the brain, leptomeninges, eyes, and spinal cord. PCNSL can develop in both immunosuppressed (IS) (HIV, organ transplant, immunosuppressive agents) and immunocompetent (IC) patients. The diagnostic confirmation is obtained from histopathology after stereotactic brain biopsy, or from vitreous or cerebrospinal fluid (CSF) cytology. In IC patients, PCNSL are mostly diffuse large B-cell lymphomas (>90%) [1,2]. In IS patients, brain lymphoma disease is essentially related to cytokine dysregulation that results from opportunistic infections with Epstein–Barr virus [3]. PCNSL incidence in IS patients greatly exceeds (about 1000 times higher) that of the general population. In IC patients, PCNSL is rare, accounting for approximately 6% of new malignant primary CNS tumors, but its incidence has increased over the past two decades, most notably in the elderly [4,5,6]. The first line treatment relies on an induction polychemotherapy based on high-dose methotrexate follow-up by a consolidation treatment with whole-brain radiotherapy or autologous stem cell transplantation in younger patients [7]. Despite advances in the management of this disease, median overall survival remains poor in the elderly population (15 months) [8,9]. In a French cohort of 1000 patients with newly diagnosed PCNSL, 25% of the elderly patients died within the first 6 months after diagnosis [9].

## 2. Brain Imaging Features at Diagnosis

### 2.1. Magnetic Resonance Imaging (MRI)

MRI is the modality of choice for the initial assessment and the follow-up of PCNSL. At diagnosis, the European Association of Neuro-Oncology (EANO) and International PCNSL Collaborative Group (IPCG) guidelines recommend T2 weighted/fluid-attenuated inversion recovery sequence (T2/FLAIR) and T1-weighted sequences pre- and post-contrast injection [10,11].

In IC, half of the lesions (50–70%) are solitary, most often located in the periventricular area (e.g., thalamus, corpus callosum and basal ganglia). The lesions usually appear isointense on T1-weighted images, slightly hypointense on T2-weighted images, and show intense homogeneous contrast enhancement without necrosis. Due to their high cellularity, brain lymphomas restrict the normal random movement of water molecules in the brain tissue, corresponding to “restricted diffusion.” This results in a hypersignal on diffusion-weighted imaging (DWI) and low apparent diffusion coefficient (ADC) values on ADC parametric maps. Vasogenic surrounding edema is usually present on T2-weighted images but classically with little mass effect relative to size [12]. On dynamic-susceptibility contrast (DSC) perfusion images, PCNSL does not show florid neoangiogenesis but a characteristic type of curve with a significant increase in signal intensity above the baseline due to massive leakage of contrast media into the interstitial space [13]. Spectroscopy can measure several different metabolites that reflect specific cellular and biochemical processes to differentiate tumor types [14]. However, PCNSL spectroscopy is not very specific and shows increased choline and decreased N-acetylaspartate (NAA), creatine (Cr), and myoinositol, which can be observed in several types of brain tumors. Nevertheless, a prominent lipid peak on spectroscopy in brain tumors without central necrosis seems to be a feature of malignant brain lymphoma [15].

**Figure 1:**
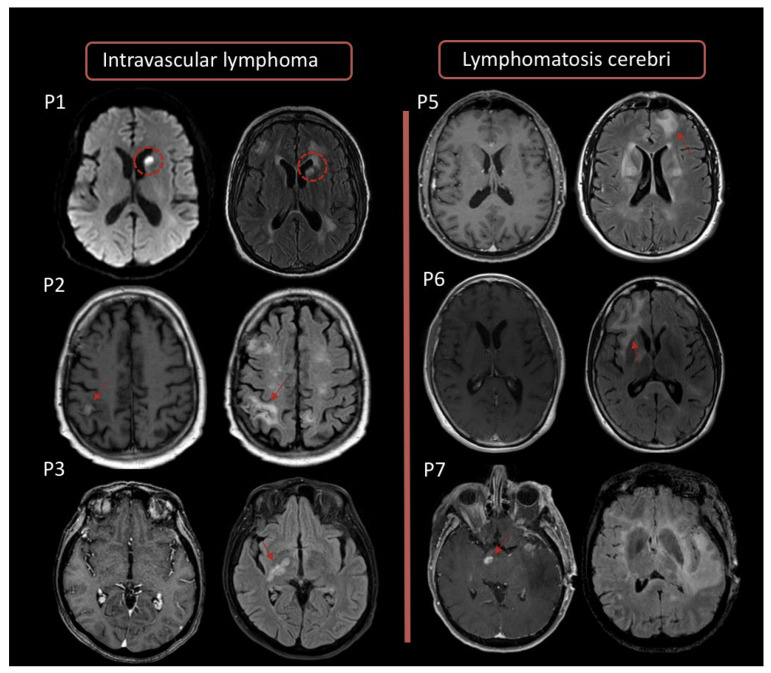
Case examples of MRI presentations of intravascular lymphoma (Patients 1, 2, and 3) and cerebral lymphomatosis (Patients 5, 6, and 7). **Left**: intravascular lymphoma presents as cortical, subcortical, or periventricular lesions with FLAIR hypersignal (2nd column) and little or no gadolinium-weighted T1+ enhancement. The diffusion-weighted image of the first patient (**P1**) shows a high hypersignal of the head of the caudate nucleus mimicking a stroke (red dotted circle). **Right**: lesions of cerebral lymphomatosis appear as infiltrating, confluent lesions with FLAIR hypersignal, in the deep and subcortical white matter (red arrows), without mass effect (4th column). T1+ gadolinium-weighted images (3rd column) show no enhancement, with the exception of the last patient (bottom right) who had both typical homogeneous enhanced lesions and an infiltrating FLAIR hypersignal.

Few studies have also reported atypical presentation such as disseminated lesions or T2-hyperintense lesions with variable diffusion restriction and no contrast enhancement [16,17,18,19] (Figure 1). In a retrospective analysis of 127 IC patient scans, Bataille et al. reported nine cases of exclusively cortical lesions mimicking a meningioma [20]. Less frequent presentations involve the eyes, leptomeninges, spine, or vascular system [21]. Only 1% of PCNSL present with spinal involvement, which typically appears as a solitary infiltrative lesion with multifocal areas of contrast enhancement. On the other hand, intravascular lymphoma is responsible for blood slowing that can lead to endoluminal thrombosis or microinfarcts.

On MRI, this subtype may appear as diffuse leukoencephalopathy or multiple small infarcts [21]. In IS patients, lesions are often multifocal, with nodular or peripheral ringlike enhancement reported in up to 75% of cases.

Although these imaging features are suggestive for PCNSL, they are not pathognomonic and several differential diagnoses may be discussed in case of suspected brain lymphoma. Therefore, confirmation of the diagnosis of PCNSL by histology or cytology is essential before treatment can be started and, if clinically possible, biopsy should be done prior to steroids as they decrease the sensitivity of the histopathological diagnosis [10]. In IC patients, the differential diagnosis includes other types of brain tumors (mainly high-grade gliomas, brain metastases), whereas progressive multifocal leukoencephalopathy and cerebral toxoplasmosis can be discussed in IS patients (Table 1) [12,22].

### 2.2. ^18^F-FDG Positron Emission Tomography (PET)

#### 2.2.1. Features at Diagnosis

**Figure 2:**
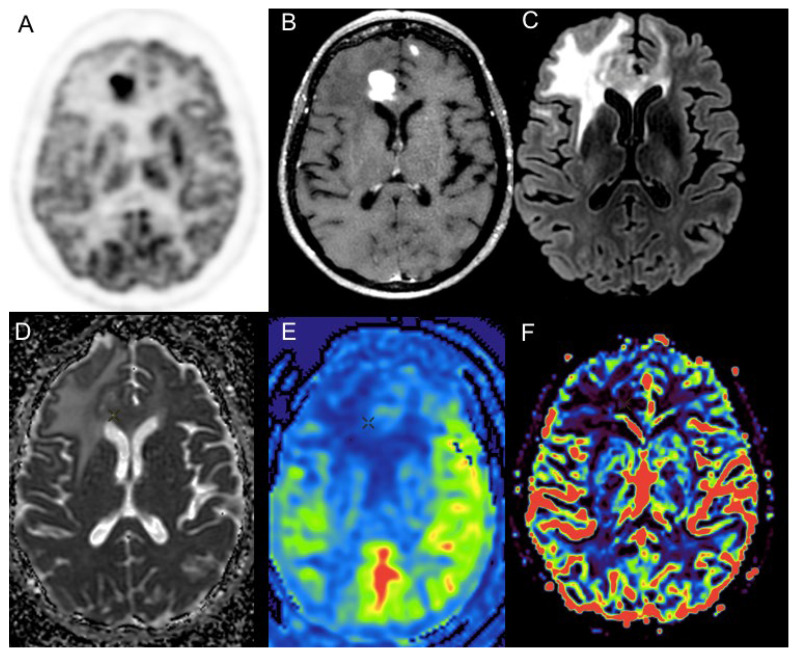
Primary central nervous system lymphoma in a 72-year-old female patient with typical PET and MRI features: high and homogeneous ^18^F-FDG PET uptake (SUVmax = 27, T/N = 2.1) (**A**); “snowball” pattern with intense and homogeneous enhancement after gadolinium enhancement (**B**); extensive hypersignal on T2-FLAIR weighted images, respecting perilesional edema and respecting the cortical ribbon (**C**). Parametric ADC map show only moderate diffusion restriction (**D**), and no hyperperfusion is detectable either on arterial spin labelling (**E**), or dynamic susceptibility contrast (**F**, rCBV parametric map) perfusion sequences.

Positron emission tomography is a molecular imaging that allows the visualization of biological targets and the detection of physiopathological processes in vivo, by targeting molecules of interest with a radiopharmaceutical radiolabeled with a short-lived radionuclide such as carbon-11 and fluorine-18. Today, ^18^F-FDG is the workhorse radiopharmaceutical used in patients with brain conditions or cancer. As has been shown for systemic lymphomas, PCNSL exhibits elevated glucose metabolism and a high avidity for ^18^F-FDG (2–3 times higher than of the healthy grey matter) due to very high cellular density with an accelerated glycolytic metabolism [19,23] (Figure 2).

In a recent meta-analysis including 486 patients from 22 studies, pooled maximum standardized uptake value (SUVmax) in PCNSL ranged from 8.4 to 27.8 with a mean of 18.1 (95% CI, 16.0–20.1) compared to 10.4 for inflammatory or malignant brain diseases other than PCNSL [24]. However, as a SUVmax cut-off value might be influenced by the model of scanner, imaging protocol or injected dose, the ratio of tumor to a contralateral mirror region of unaffected brain tissue (T/N) is preferentially analyzed. Gupta et al. showed that a T/N ratios cut-off at 1.66 led to a PPV and NPV of 90% (95% CI: 79–96%) and 69% (95% CI: 54–81%) for differentiating CNS lymphomas from non-lymphomas diseases [25].

A limitation that has been raised for PCNSL detectability with ^18^F-FDG-PET is the potential impact of corticosteroids [26,27,28]. Corticosteroids can induce apoptosis of lymphoma cells very rapidly and it has been recognized that pre-administration of corticosteroids can significantly shrink the size of the tumor and even induce tumor disappearance on MRI and histological evaluation [29]. However, the impact of steroids on ^18^F-FDG uptake remains unclear. In a small cohort, Rosenfeld et al. first described a difference in SUV between steroid-treated (*n* = 7) and untreated cases (*n* = 3) [28]. Yamagashi et al. confirmed a negative correlation between T/N and cumulative corticosteroid dose in a retrospective study of 19 patients. However, these differences did not reach statistical significance in either study [28,30].

**Figure 3:**
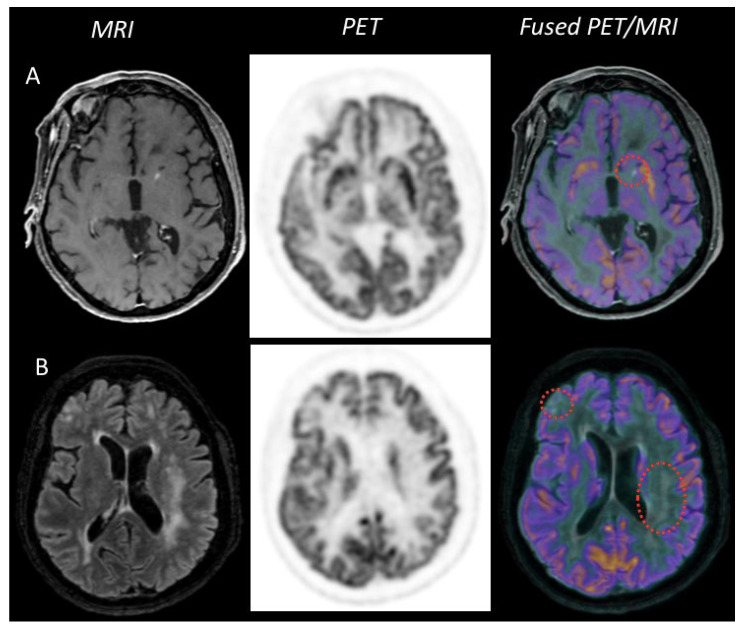
Example case of PET-MRI images of an intravascular lymphoma. Gadolinium-weighted T1+ imaging (**A**) showed a small enhanced punctiform lesion in the medial part of the left striatum, with no metabolic uptake on ^18^F-FDG PET images. The cortical and subcortical lesions in FLAIR hypersignal (**B**) did not show uptake with ^18^F-FDG either.

**Figure 4:**
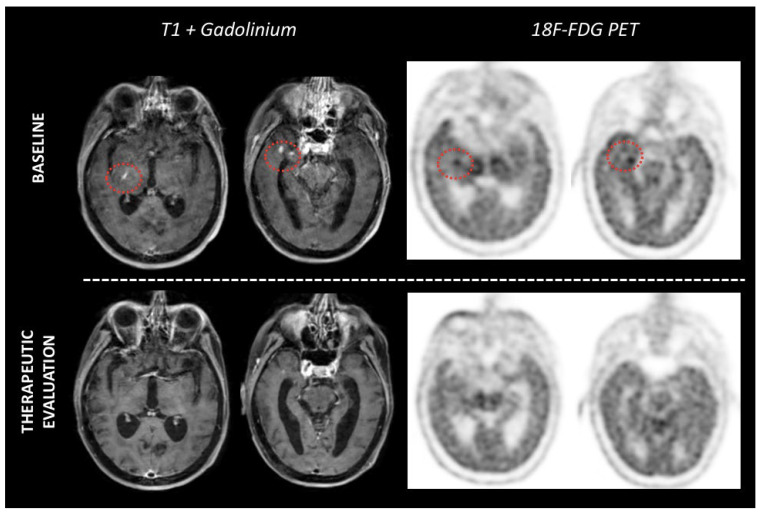
Example case of PET-MRI images of a lymphotamosis cerebri before and after Methotrexate therapy. Baseline images (**top line**) showed small nodular enhanced lesions anterior to the temporal horn of the right ventricle and a linear contrast-enhanced lesion at the posterior arm of the right internal capsule. ^18^F-FDG PET images revealed strong uptake consistent with these lesions. After three cycles of methotrexate treatment (**bottom line**), MRI images showed a partial response, while ^18^F-FDG PET images had already normalized.

Of note, ^18^F-FDG avidity appears to be independent of tumor size [31]. In addition, atypical MRI presentation forms of PCNSL (disseminated lesions with faint enhancement, lack of enhancement, and ring-shaped enhanced lesions) have been described with significantly lower uptake values than typical PCNSL [32] (Figure 3 and Figure 4). Recently, Kim et al. suggested that low ^18^F-FDG avidity was more frequently related to negativity for MUM1 expression, a protein known to be a crucial regulator of B-cell development and tumorigenesis [33].

In IC patients, the main differential diagnosis of PCNSL is high-grade glioma (HGG), the most prevalent and aggressive primary malignancy of the brain parenchyma. To distinguish between these two entities, brain imaging techniques are valuable tools: on the one hand, to help clinicians decide between stereotactic biopsy in case of suspected lymphoma or extensive surgery in case of suspected HGG; on the other hand, to quickly decide on therapeutic management if the patient is recused for invasive procedure.

However, the MRI presentation of HGG can mimic that of PCNSL and vice versa: a “homogeneous pattern” with a “butterfly pattern through the corpus callosum” can be observed in true HGG whereas confirmed PCNSL in IC patients may show annular enhancement with central necrosis, normally seen in HGG [12,16]. Literature has shown good diagnostic performance of ^18^F-FDG PET-CT in this setting, as PCNSL is known to have higher ^18^F-FDG uptake than HGG [34,35]. Some studies have proposed SUV_max_ cut-off ranging from 12 to 15 to confirm the diagnosis of PCNSL [27,36]. A recent study of 65 immunocompetent patients confirmed that T/N was significantly lower in HGG than in PCNSL. Sensitivity and specificity of T/N vary respectively between 90–100% and 75–87%, depending on the threshold used by the different teams [34,37,38]. In contrast, no added value was reported for metabolic tumor volume or total lesion glycolysis [37]. In addition, the combination of high T/N (cutoff value of 2.23) on ^18^F-FDG PET and low maximal tumor blood flow on ASL (cutoff value of 2.07) has been described to improve discrimination between PCNSL and HGG (accuracy 99.1%, sensitivity 95%, and specificity 96.4%) [34].

To further improve radiological diagnosis, machine learning has recently emerged as an important tool. Petersen et al. conducted a meta-analysis of 23 papers, including 3 papers on ^18^F-FDG, that published machine learning-based classification algorithms to distinguish between HGG and PCNSL [39]. The machine learning models appear to have high accuracy as the algorithms were able to replicate the results of a senior subspecialty-trained radiologist. Using radiomic approach, Kong et al. recently reported AUC as high as 0.971–0.998 for the selected features [40].

In IS patients and especially in HIV-infected patients, CNS lymphoma and toxoplasmosis are the two most common brain disease. While the distinction between these two entities is critical because of the dramatic change in patient management, their MRI features may overlap. Although considered the gold standard, histopathologic confirmation is rarely available and diagnosis relies on an anti-toxoplasma treatment test that may delay initiation of adequate chemotherapy in true CNS lymphoma. Single-photon emission computed tomography (SPECT) using thallium-201 has historically been used for differential diagnosis between tumor and non-neoplastic lesions, because thallium-201 accumulates in actively dividing cells via a transport promoted by an adenosine triphosphate cell membrane active pump but not in infectious process [41]. In a meta-analysis of 667 patients from 18 studies, Yang et al. found a respective pooled sensitivity and specificity for thallium-201 SPECT of 92% and 84% [42].

**Figure 5:**
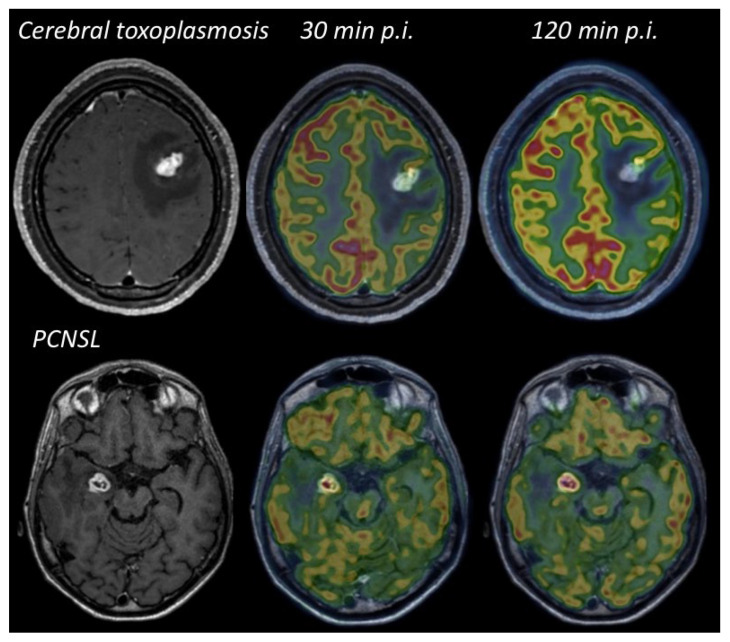
Illustrative case of the value of delayed acquisition of ^18^F-FDG PET to differentiate infection from malignancy in an IS patient. The first patient was a 19-year-old female with AIDS disease, while the second patient was undergoing immunosuppressive therapy for renal transplantation. The ^18^F-FDG PET scan was performed for both patients at 30 and 120 min after injection (p.i.). In the first case, tracer uptake was very low at 30 min p.i. and decreased with time (SUVmax = 4.7 and 3.2; T/N = 0.3 and 0.2). The patient was diagnosed with cerebral toxoplasmosis by serological evaluation. In the second case, the lesion showed a progressive increase in metabolic activity over time compared with normal tissue (SUVmax = 5.9 and 11.2; T/N = 1.5 and 1.7) and brain biopsy confirmed brain lymphoma.

^18^F-FDG has also been extensively evaluated [43,44,45,46,47,48]. All studies revealed high accuracy of this technique with sensitivities close to 100% and specificities ranging from 75% to 100% i.e., high metabolic activity for CNS lymphoma versus normal to reduced intralesional metabolic activity relative to adjacent brain parenchyma for cerebral toxoplasma [43,44,45,48] (Figure 5).

In summary, while ^18^F-FDG brain PET appears to be highly reliable for the differential diagnosis of brain lesions in IS patients, its potential role in IC patients needs to be further analyzed, especially in the new era of artificial intelligence algorithm applied to PET.

**Table 1:** MRI and PET characteristics of PCNSL and its main differential diagnoses [16,49,50,51,52,53].

		MRI								PET	
		T1	T1+Gd	T2W/FLAIR	DWI	SWI	ADC	Spectroscopy	rCBV	^18^F-FDG	^11^C-MET
Biophysical Features		Morphological Features	BBB Disruption	Vasogenic Edema	Cellular Density	Detection of Hemosiderin, Ferritin and Calcium	Cellular Restriction	Detection of Brain Metabolites	Microvascular Blood Volume	Glucidic metabolism	Amino-Acid Analog
**IC patients**	PCNSL IC	Iso or hypointense Uni or multifocal Profound or periventricular	Homogeneously enhancing parenchymal mass	Tumor is in hyposignal compared to the hypersignal of the adjacent edema FLAIR	Hyperintense	Rare microhemorrhage and calcification	Very low (lower than GBM)	Cho/Cr ⬈ Lipid ⬈ NAA ⬊ (less marked than in glioblastoma)	Low Typical perfusion curve returning above the baseline	High uptake	High uptake
	Gliobastoma	Hypo to isointense Central hemispheric white matter	Heterogeneous enhanchement with central necrosis	Hyperintense surrounded by vasogenic edema	Hyperintense in the solid portion	Frequent small hemorrhages	Low	Cho/Cr ⬈ Lactate ⬈ Lipids ⬈ NAA ⬊	High	High uptake (but less pronounced than in PSCNSL)	High uptake
**IS patients**	PCNCL ID	Iso or hypointense or Multifocal lesions Basal ganglia and corpus callosum	Nodular, or ringlike patterns	Hypointense to slightly hyperintense Small amount of edema and mass effect	Variable	Spontaneous hemorrhage more frequent than for PCNSL IC	Usually rADC <1.6 but overlapping ratio with toxoplasma lesions	Choline ⬈ NAA ⬊	Low	High uptake	High uptake
	Toxoplasmosis	Iso to hypointense Multifocal Basal ganglia, corticomedullary junction of the cerebral hemispheres	Ringlike or nodular enhancement patterns	Low hypointense to hyperintense Large edema and mass effect	Variable	Occasional hemorrhages	Usually rADC >1.6 but overlapping ratio with PCNSL	Choline mild ⬈ NAA ⬊ Lactate ⬈ Lipids ⬈	Low	Hypometabolic	Low to high uptake
	Progressive Multifocal Leukoencephalopathy	Hypointense Multifocal and asymetric involvement Subcortical white matter and centrum semi ovale	Non nenhancing or, rarely, mildly enhancing.	Hyperintense Multiple small punctate lesions outside the main PML lesions	Central core with low signal surrounded by a rim of high signal intensity	Typical leukocortical band/rim	Variable/Low ADC reflects active lesions	Choline ⬈ NAA ⬊ Lactate ⬈ Lipids ⬈	Low	Low uptake	Low to high uptake

Gd: gadolinium, FLAIR: Fluid-attenuated inversion recovery, DWI: diffusion-weighted images, SWI: susceptibility-weighted images, ADC: apparent diffusion coefficient, rCBV: relative cerebral blood volume, For spectroscopy: choline (Cho): marker of cell membrane synthesis. lactate: anaerobic glycolysis. N-acetylaspartate (NAA): neuronal viability. Creatinine (Cr): cell density. Lipids: cell necrosis. PML: progressive multifocal leukoencephalopathy.

#### 2.2.2. Outcome Prediction

PCNSL has poor prognosis. To date, the only established prognostic factors are age and functional status at diagnosis [9]. For therapeutic trials, two multipoint scoring scales including clinico-biological factors have been proposed, the International Extranodal Lymphoma Study Group (IELSG) scale, and the Memorial Sloan-Kettering Cancer Center (MSKCC) scale [54,55]. Recently, other biological factors or molecular characteristics of tumors have been identified. For example, it has been reported that high protein levels of interleukin (IL)-10 at diagnosis or co-expression of MYC/BCL2 in tumors correlate with low overall survival (OS) and progression-free survival (PFS) [56,57]. In addition, many prognostic factors have been proposed in MRI, including multifocal lesions, high tumor volume, or subtentorial involvement, but with controversial results [58,59]. Some also analyzed more advanced parameters, i.e., diffusion and perfusion MRI sequences, with promising results for ADC parameters or blood plasma volume (Vp) and volume transfer constant (Ktrans) [60,61]. However, none of these results have been corroborated by studies with large patient cohorts.

As mentioned previously, CNS lymphoma is characterized by high ^18^F-FDG uptake. Several studies have explored the role of intensity of ^18^F-FDG uptake or hypermetabolic volume on pretreatment PET, as prognostic markers. Varied indexes have been proposed but they have not been always corroborated (Table 2). A poorer survival was associated with high SUVmax (>12) in a series of 17 IC patients [62]. Kasenda et al. developed a visual scale based on the tumor SUVmax normalized to the normal cerebellar uptake (MILAS for Metabolic Imaging Lymphoma Aggressiveness Scale). They found a negative correlation between increased MILAS (greater than 3 corresponding i.e., ^18^F-FDG uptake of the tumor 200% higher than that of the cerebellum) and PFS (54.7 vs. 3.8 months) [63]. Other studies have emphasized the potential prognostic role of total lesion glycolysis (TLG) or metabolic tumor volume (MTV) [64,65]. For example, PFS and OS were significantly shorter in patients with MTV ≥ 9.8 cm^3^ and TLG ≥ 94 g/mL × cm^3^ in a cohort of 52 patients [65]. More recently, Krebs et al. suggest that adding SUVmax values and volumes of all lesions (up to five) may predict response at the end of treatment and are related to the risk of disease progression or death [66]. In conclusion, high ^18^F-FDG uptake and large metabolic tumor volume seem to be good predictors of poor prognosis; however due to the lack of consensus and the limited number of patients in each study, neither of these parameters is yet applicable in daily routine.

## 3. Role of ^18^F-FDG PET in the Management of PCNSL

### 3.1. Systemic Assessment

Identification of systemic disease in suspected brain lymphoma is pivotal to treatment decisions, as chemotherapy regimens vary between systemic lymphoma with CNS involvement and PCNSL, which requires additional methotrexate therapy. Historically, staging evaluation included CT scan, bone marrow biopsy, and testicular ultrasound in men [67].

**Figure 6:**
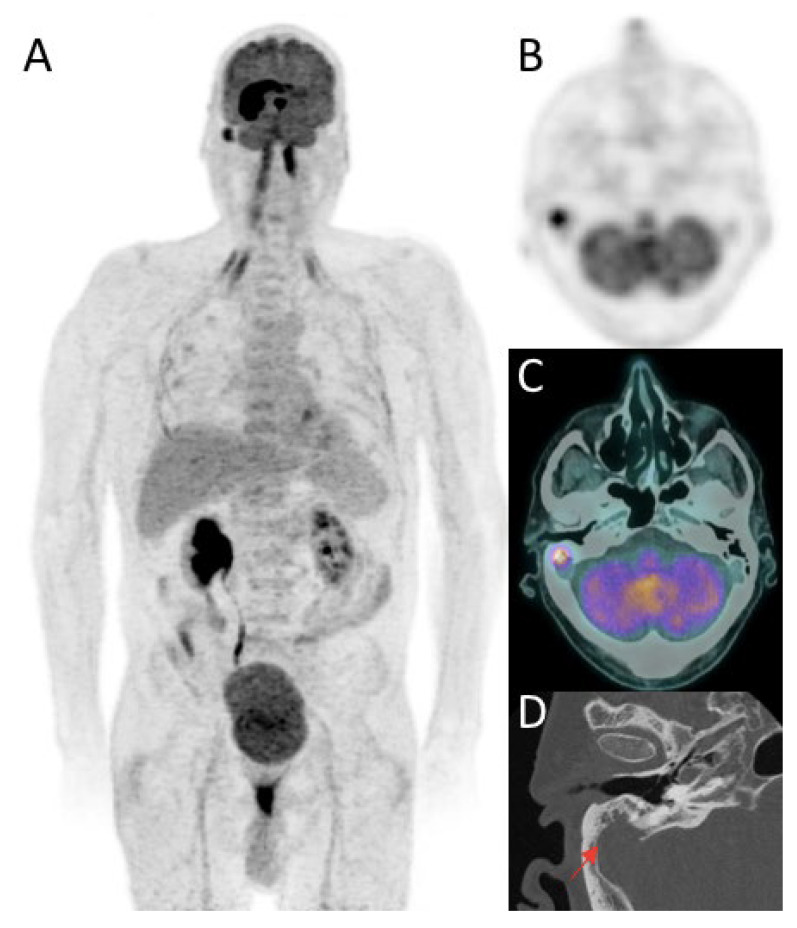
^18^F-FDG PET/CT reveals systemic involvement in the right mastoiditis in a patient with right temporal and paraventricular brain lesion highly avid for ^18^F-FDG. **A**: Maximum intensity projection (MIP) of ^18^F-FDG PET images. PET images showed an hypermetabolic right mastoid process filling: SUVmax at 8.8 (**B**: axial PET images, **C**: axial PET/CT fusion). Imaging was completed by a dedicated thin-section CT scan which revealed irregular focal bone lysis of the right mastoid (**D**). Histological examination confirmed a B-cell lymphoma proliferation.

First studies showed low additional value of body ^18^F-FDG PET to detect systemic involvement, found in only 2% of patients [68]. With the emergence of new PET-CT systems, literature has shown that body PET could detect systemic disease in up to 10% of patients [25,69]. Several studies have reported superior diagnostic performance of whole-body ^18^F-FDG PET-CT compared to whole-body CT (4.9% vs. 2.5%) [31,68,69,70]. Advantage of PET is to identify malignant foci outside the “chest-abdomen-pelvis” (CAP) area, investigated by CT [71] (Figure 6). A recent meta-analysis on 1040 patients, reported systemic lymphoma lesions in upper and lower limbs bones, lymph nodes, adrenal gland, soft tissue, spleen, liver, jejunum, testicle, thyroid gland, and nose [72]. Although the increase in false-positive ^18^F-FDG PET results has been blamed for unnecessary interventions, PET also enables detection of incidental secondary malignancies (1.5% to 3.1%) with significant therapeutic impact [72].

Today, the role of whole-body ^18^F-FDG-PET in the initial diagnostic of PSCNL to rule out systemic involvement has been set in stone by several expert societies and PET should be prescribed as first-line when its realization does not compromise prompt disease management [10,11,67,73].

### 3.2. Therapeutic Evaluation

Over the past decades, several therapeutic trials have proposed new treatment regimens for patients with PCNSL [74]. Currently, multidrug therapy including high-dose methotrexate is a standard of care and is combined with whole-brain radiotherapy or autologous stem-cell transplantation in young patients. Because of the high risk of neurotoxicity, whole brain radiotherapy is no longer recommended for patients older than 60 years [75].

Today, treatment follow-up is based on the International PCNSL Collaborative Group (IPCG) radiographic response criteria, which recommends gadolinium-enhanced MRI performed approximately every 2 months during active treatment [67]. However, this classification includes an unconfirmed complete response, because of the inability of MRI to distinguish residual tumor nidus from biopsy-related scar tissue [67,76]. In addition, the early onset of recurrence after treatment in patients with a complete response on MRI raises the question of the performance of MRI in assessing residual disease. Recently, Van der Meulen et al. showed that there were no significant differences in PFS and OS between patients with a complete or partial MRI response at the end of protocol treatment in a phase III randomized controlled trial of 199 patients with PCNSL [77].

**Figure 7:**
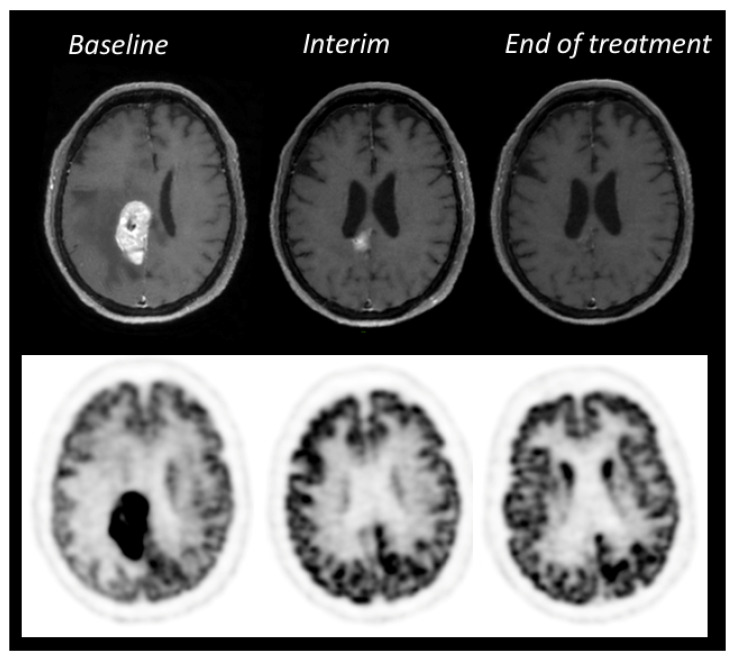
Therapeutic ^18^F-FDG PET-MRI evaluation of PCNSL in an immunocompetent patient treated with R-MPVA chemotherapy (Rituximab, Methotrexate, Procarbazine, Vincristine, Cytarabine). Two months after the beginning of the treatment, MRI showed a partial response (81% decrease of the enhanced MRI volume on 3DT1-gadolinium sequence) while PET was already negative. Follow-up showed a complete response on both MRI and PET at the end of treatment and the patient was disease free after 3 years. This case illustrates the potential role of interim PET as an early prognostic biomarker.

While the role of interim ^18^F-FDG PET has been well established in aggressive systemic lymphomas, few studies have examined its value in PCNSL. Birsen (*n* = 25 patients), Jo (*n* = 66), and Palmedo (*n* = 8) et al. agree on the prognostic impact of interim PET on the end-of-treatment response with a high negative predictive value (94.74% to 100%) [78,79,80]. In addition, they showed that PET was an earlier biomarker of response than morphological MRI. However, it is still unclear when PET should be performed to stratify patients according to their prognosis between interim and final evaluation [78,79,80,81,82]. Further prospective trials in large cohorts are therefore needed to establish PET in the therapeutic management of patients with PCNSL. A prospective study exploring ^18^F-FDG PET in treatment responses in CNS lymphoma is currently underway in France (NCT03582254) to address the role of ^18^F-FDG PET in the management of CNS lymphoma during treatment (Figure 7).

**Figure 8:**
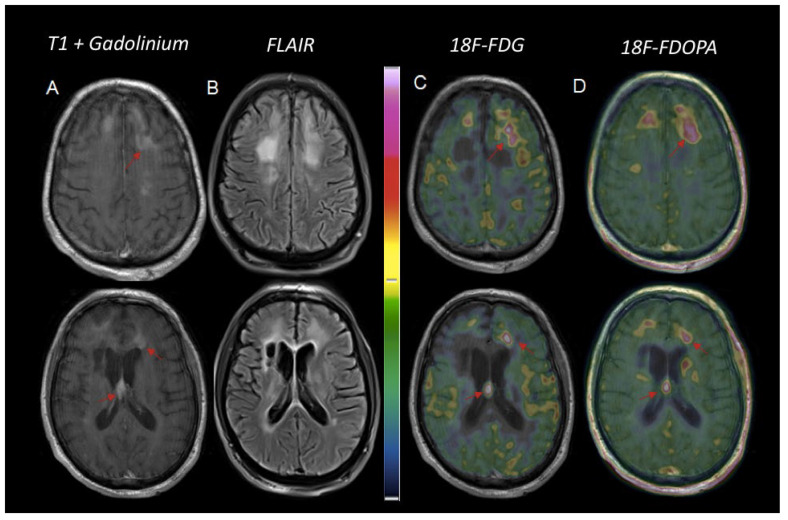
Illustrative case of suspicion of pseudoprogression versus progression in a 57-year-old man with IC PCNSL treated with immunotherapy (Pembrolizumab). MRI images showed suspicious bi-frontal and interventricular contrast-enhanced lesions on post gadolinium T1-weighted MRI, (**A**) and a T2-FLAIR hypersignal (**B**). Two enhanced-lesions located in the septum pellucidum and in front of the left frontal horn of the lateral ventricle, extending through the surrounding white matter, were highly hypermetabolic on ^18^F-FDG PET (**C**: axial PET) and showed strong uptake on ^18^F-FDOPA PET (**D**: axial PET): tumoral to normal contralateral tissue ratio (T/N) = 3.9 and 1.9 respectively, with ^18^F-FDG versus 2.7 and 2.2 with ^18^F-FDOPA. A third enhanced-lesion in the right frontal lobe showed milder uptake both on ^18^F-FDG and ^18^F-FDOPA PET (T/N = 1.5 and 1.8, respectively), but was considered suspicious due to its localization in white matter. Follow-up MRI confirmed the diagnosis of PCNSL relapse.

Recently, the role of immune evasion and suppression of the immune microenvironment has been revealed as a key element of the pathogenesis of PCNSL. Therefore, new therapeutic trials using immunotherapy agents are currently underway [83]. On MRI, these agents may be responsible for an effect called pseudoprogression, related to changes in BBB permeability: contrast enhancement may increase or even appear under treatment while there is no real tumor progression, and follow-up shows a normalization of these images without therapeutic changes [11]. While these phenomena have been extensively studied in glial pathologies on PET, no study has yet reported relevant elements on this subject, to our knowledge in PCNSL (Figure 8).

## 4. Others PET Tracers in PCNSL

Others PET tracers have been evaluated for characterization and therapeutic evaluation of PCNSL. The most analyzed is ^11^C-Methionine (^11^C-MET), an amino acid tracer correlated with cell proliferation activity, which benefits from a low uptake in normal brain compared to ^18^F-FDG. In pre-therapeutic evaluation, the literature has shown good agreement between ^18^F-FDG and methionine tracers but without additional diagnostic or prognostic value for the latter [84,85]. ^11^C-MET could also be useful for early detection of non-responders, as it has been described that a high uptake level at the interim assessment (after four cycles of methotrexate) was associated with decreased free survival [86]. Few studies on small cohort have suggested a potential additional role of ^11^C-MET, for example to stratify patients according to the immunohistochemical subtypes or for differential diagnostic between CNSL and HGG based on kinetic analysis [87,88]. However, the main limitation of ^11^C-MET is the need for an on-site cyclotron due to the short 20-min half-life of this radiotracer.

In addition to amino acid analogues, other PET tracers studied for CNS lymphomas are ^68^Ga-Pentixafor and ^18^F-Fludarabine. ^68^Ga-Pentixafor specifically targets CXCR4 receptors that are overexpressed by lymphoma, leukemia, and myeloma [89]. In two small pilot studies, Starzer et al. (*n* = 7) and Herhaus et al. (*n* = 8) showed 100% accuracy for detection of PCNSL between MRI and ^68^Ga-Pentixafor, with high contrast between lesions and normal brain background uptake. In addition, lesions with lower uptake of the CXCR4 tracer were associated with a better response to standard methotrexate-based therapy, suggesting a potential role for this new tracer for therapeutic follow-up applications [89,90]. However, the additional value of ^68^Ga-Pentixafor remains questionable, especially for atypical PCNSL, because this tracer is dependent on the breakdown of the blood-brain barrier. In preclinical studies, Hovanishyan et al. showed that ^18^F-Fludarabine (^18^F-FDB), a PET radiotracer version of the drug fludarabine that binds equilibrium nucleoside transporters (ENT1), could distinguish glioblastoma from CNS lymphomas, without being biased by activated macrophages and other inflammatory cells [91]. ^18^F-FDB showed high uptake in CNS lymphomas, while the tracer was rapidly cleared from glioma cells. These results have recently been confirmed in a first-in man study using PET with ^18^F-FDB [92]. Early results on ^18^F-FDB also showed that the tracer uptake could be independent of BBB leakage, which should allow metabolic characterization of atypical PCNSL [91,93].

## 5. Conclusions

^18^F-FDG is the most widely used radiotracer in this disease because of its high uptake by lymphoid cells and its easy and inexpensive access.

Its role in systemic assessment at initial diagnosis has been widely established by expert society over the past decade. Several studies have evaluated its prognostic role during follow-up, but at present, the available data are from small, mostly retrospective series. Furthermore, in the new era of machine learning, future biomarkers are likely to be a combination of parameters from different imaging techniques, and nuclear physicians and neuroradiologists will need to cooperate closely to have a real impact on clinicians’ management decisions.

In addition, several other radiotracers have been proposed, including ^11^C-MET, ^68^Ga-Pentixafor, and ^18^F-Fludarabine. Further prospective studies are needed to confirm their potential role in the management of PCNSL.

## Figures and Tables

**Table 2:** ^18^F-FDG PET Biomarkers of poor PFS.

Biomarkers	Threshold	Analysis	HR	95% CI	*p*	REF
SUVmax	≥12	Univariate			<0.05	Kawai [62]
MILAS	>3 i.e.	Multivariate	1.46	1.10–1.94	0.010	Kasenda [63]
MTV	≥9.8 cm^3^	Mutlivariate	5.35	1.89–12.8	0.037	Albano [65]
TLG	≥94	Multivariate	4.54	1.37–11.6	0.045	Albano [65]
	>227	Univariate			0.02	Okoyucu [64]
Sum SUVmax	-	Multivariate	1.09	1.04–1.14	<0.001	Krebs [66]
